# Oral–Gut Microbiota, Periodontal Diseases, and Arthritis: Literature Overview on the Role of Probiotics

**DOI:** 10.3390/ijms24054626

**Published:** 2023-02-27

**Authors:** Martina Ferrillo, Amerigo Giudice, Mario Migliario, Filippo Renó, Lorenzo Lippi, Dario Calafiore, Nicola Marotta, Roberto de Sire, Leonzio Fortunato, Antonio Ammendolia, Marco Invernizzi, Alessandro de Sire

**Affiliations:** 1 Dentistry Unit, Department of Health Sciences, University of Catanzaro “Magna Graecia”, 88100 Catanzaro, Italy; 2 Dentistry Unit, Department of Translational Medicine, University of Eastern Piedmont, 28100 Novara, Italy; 3 Innovative Research Laboratory for Wound Healing, Health Sciences Department, University of Eastern Piedmont, 28100 Novara, Italy; 4 Physical and Rehabilitative Medicine, Department of Health Sciences, University of Eastern Piedmont, 28100 Novara, Italy; 5 Translational Medicine, Dipartimento Attività Integrate Ricerca e Innovazione (DAIRI), Azienda Ospedaliera SS. Antonio e Biagio e Cesare Arrigo, 15121 Alessandria, Italy; 6 Physical Medicine and Rehabilitation Unit, Department of Neurosciences, ASST Carlo Poma, 46100 Mantova, Italy; 7 Physical Medicine and Rehabilitation Unit, Department of Medical and Surgical Sciences, University of Catanzaro “Magna Graecia”, 88100 Catanzaro, Italy; 8 Gastroenterology Unit, Department of Clinical Medicine and Surgery, University Federico II of Naples, 80126 Naples, Italy

**Keywords:** oral microbiome, gut microbiota, periodontal disease, gastrointestinal microbiome, osteoarthritis, knee osteoarthritis, inflammaging, gut dysbiosis, diet, probiotics

## Abstract

Periodontal diseases are oral inflammatory diseases affecting the tissues supporting and surrounding the teeth and include gingivitis and periodontitis. Oral pathogens may lead to microbial products spreading into the systemic circulation and reaching distant organs, while periodontal diseases have been related to low-grade systemic inflammation. Gut and oral microbiota alterations might play a role in the pathogenesis of several autoimmune and inflammatory diseases including arthritis, considering the role of the gut–joint axis in the regulation of molecular pathways involved in the pathogenesis of these conditions. In this scenario, it is hypothesized that probiotics might contribute to the oral and intestinal micro-ecological balance and could reduce low-grade inflammation typical of periodontal diseases and arthritis. This literature overview aims to summarize state-of-the-art ideas about linkages among oral–gut microbiota, periodontal diseases, and arthritis, while investigating the role of probiotics as a potential therapeutic intervention for the management of both oral diseases and musculoskeletal disorders.

## 1. Introduction

Periodontal diseases are oral inflammatory diseases affecting the tissues supporting and surrounding the teeth and include gingivitis and periodontitis [1,2]. Furthermore, gingivitis is associated with bleeding, swollen gums, and pain, whereas periodontitis is related to the loss of periodontal attachment and supporting bone [3]. The latter is often considered a “silent disease” due to the absence of symptoms that characterize the clinical presentation. However, if untreated, the inflammatory condition of periodontitis can lead to tooth loss, with a consequent impairment in mastication function, esthetics, self-confidence, and quality of life [4,5]. The prevalence of periodontal diseases is estimated to range from 20% to 50% worldwide, emerging as the 11th most prevalent condition in the world as reported by the Global Burden of Disease Study of 2016 [6]. Therefore, this detrimental condition is currently considered as a global health problem [7,8].

In this scenario, the first step is understanding the mechanisms underpinning the etiopathogenesis of periodontal diseases, considering that the local inflammatory response might be perpetuated by several oral pathogens (e.g., *A. actinomycetemcomitans*, *P. intermedia*, *P. gingivalis*, *T. denticola*, *F. nucleatum*, and *T. forsyth*) [9,10,11].

Periodontal diseases have been shown to be related to low-grade systemic inflammation, potentially driven by several inflammatory mediators [9,10,12,13,14]. In this context, recent studies showed that patients with periodontal diseases might be characterized by higher circulating levels of C-reactive protein (CRP), fibrinogen, neutrophils, and indirect systemic inflammatory markers, such as tumor necrosis factor (TNF) and Interleukin (IL) 1, 6, and 8 [15,16,17].

High levels of bacteria present in the dysbiotic biofilm in periodontitis might also play a role in the pathogenesis of autoimmune diseases [18,19,20]. In this context, recent studies reported a link between periodontitis and rheumatoid arthritis (RA), considering the higher prevalence of RA in these patients and the correlation between the severity of arthritis and periodontitis [19]. Indeed, it was reported that *P. gingivalis* has been implicated in the generation of anticyclic citrullinated peptide antibodies (ACPAs), which are recognized as diagnostic and prognostic biomarkers for RA patients [20]. Moreover, Zhou et al. [19] suggested that the downregulation of IL-10 could represent the key mechanism by which periodontitis may promote RA.

Several scientific studies showed that gut and oral microbiota alterations might play a role in the pathogenesis of several autoimmune and inflammatory diseases [21,22,23,24].

**Figure 1:**
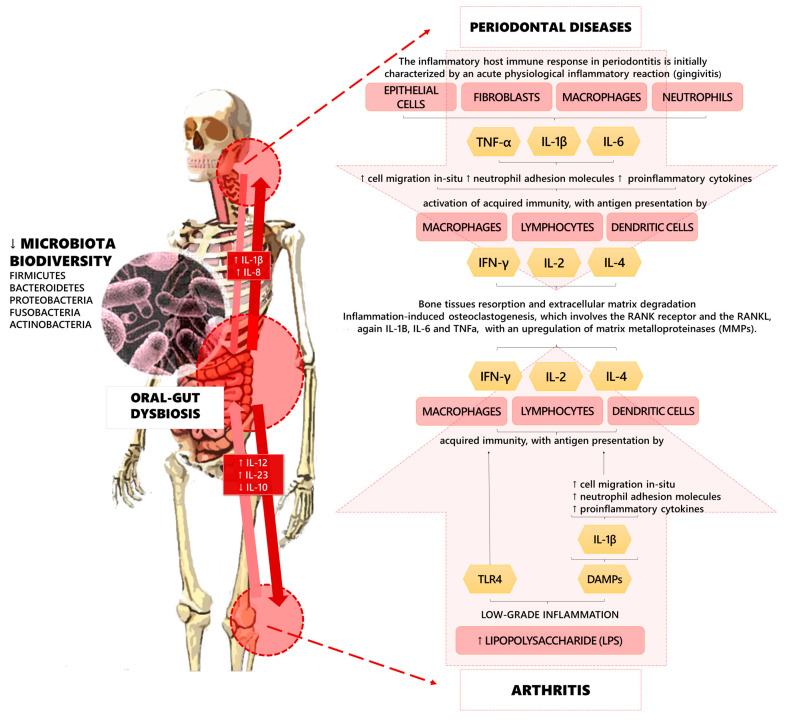
Pathogenic pathways of the linkages among oral–gut dysbiosis, periodontal diseases, and arthritis.

However, recent research has focused on the implications of the microbiota on musculoskeletal health, highlighting the role of the “gut–bone axis” and the “gut–joint axis” in the regulation of molecular pathways involved in the pathogenesis of these detrimental conditions [25,26,27,28]. Furthermore, gut microbial dysbiosis might promote musculoskeletal disorders through the intestinal absorption of vitamin K, calcium, pyridoxal phosphate, pantothenic acid (B5), cobalamin (B12), biotin (B7), folate (B9), thiamine (B1), niacin (B3), and tetrahydrofolate. Moreover, it has been proposed that osteoclasts activity might be indirectly stimulated by gut microbiota via serum levels of insulin-like growth factor 1 (IGF-1) [29,30,31] (see Figure 1 for further details).

Although the pathophysiological mechanisms underpinning the interactions among oral–gut microbiota have still not been characterized in detail, it has been hypothesized that dietary supplements including probiotics could contribute to the oral and intestinal micro-ecological balance [26,32,33,34]. However, to date, there is still a large gap of knowledge about the optimal management of oral and gut dysbiosis.

Therefore, the present literature overview aims to summarize the current scientific evidence on correlations among oral–gut microbiota, periodontal diseases, and arthritis, exploring the role that probiotics might play as a therapeutic intervention for oral diseases and musculoskeletal disorders.

## 2. Oral–Gut Microbiome and Periodontal Diseases

Microbiota includes microbial communities colonizing the mucosae (such as the intestinal tract, reproductive organs, and the respiratory tract) and the skin, for a total of more than 100 trillion microbial cells that encode 100-fold more unique genes than the human genome [35,36,37]. Microbiota colonization appears in the early years of life and changes rapidly, until it becomes unique for each person, then remains relatively stable in adulthood [38]. The oral cavity is one of the most complex ecosystems in the body due to its repeated interaction with the external environment, as well as containing several different microbial habitats, both hard tissues (i.e., the teeth) and soft tissues (i.e., the buccal mucosa, the tongue, the soft and hard palates, and the gingiva) and their respective interfaces (i.e., the supragingival and subgingival margins) [39].

The oral microbiome is estimated to be the second most divergent and abundant after the gut microbiota, considering that it is mainly composed of bacteria, viruses, fungi, protozoa, and archaea; indeed, in the oral cavity of humans, 700 bacterial species, belonging to 185 genera and 12 phyla, have been identified [39]. The oral microbiome includes different phyla consisting of Firmicutes (including *Streptococcus*), Bacteroidetes (strongly represented by *Prevotella*), Proteobacteria, Fusobacteria, and Actinobacteria [40]. These bacteria commonly coexist and thrive by forming a biofilm and living in a symbiotic state of co-aggregation, thus maintaining the homeostasis of the oral ecosystem; furthermore, it should be noted that the plaque biofilm can create an adequate balance among the pathogens and commensals and is highly resistant to the environmental stimuli [41,42,43].

Furthermore, fungi are an integral part of a healthy oral microbiota, where commensal fungi entertain a multitude of synergic or antagonistic interactions with bacteria. It is estimated that more than 100 species of fungi colonize the oral cavity (e.g., *Candida* species, *Cladosporium*, *Aureobasidium*, *Saccharomyces*, *Aspergillus*, *Fusarium*, and *Cryptococcus*), and only in immunocompromised subjects or specific conditions (especially drug abuse) can they become opportunistic pathogens [44,45].

Interestingly, the oral microbiome might be affected not only by the overall health condition of the host but also by environmental and behavioral factors including oral hygiene, nutrition, smoking, and mechanical stress [46]. In particular, the regular consumption of beverages and food with elevated levels of polyphenols (e.g., tea, cranberry, and almond) have been shown to inhibit some oral pathogenic bacteria [47,48]. Meanwhile, Esberg et al. reported that some species, including *Actinomyces*, *Bifidobacterium*, *Veillonella*, and *Streptococcus* (e.g., *S. wiggsiae*, *S. mutans*, and *S. sobrinus*) were frequently associated with high sucrose intake [49].

The gut microbiota shows several differences compared to the oral one, including pH and O_2_ tension, host secretions, substrate availability, and digest flow rates [50]. It should be noted that the gastric tract (median pH 1.4) is mainly colonized by Actinobacteria, Bacteroidetes, Firmicutes (including *Streptococcus*), and Proteobacteria (which include *Helicobacter pylori*) in healthy subjects [51]. The large intestine hosts the most abundant microbial community, probably because of the slow flow rates and the neutral-to-mildly-acidic pH [52], while the main gut bacterial phyla are the Firmicutes (including *Clostridium*, *Enterococcus*, *Lactobacillus*, and *Ruminococcus* genera), Bacteroidetes (including *Prevotella* genera), Actinobacteria, Proteobacteria, and Fusobacteria [37]. However, several pathological conditions might occur in response to the loss of the balance within a human-associated gut microbiota; such gut dysbiosis might be closely related to inflammatory bowel disorders (e.g., Crohn’s disease), esophagitis, Barrett’s esophagus, vaginitis, type 2 diabetes, arthritis, autism, neurodegenerative diseases, and cancer [24,35,53,54,55]. Furthermore, gut microorganisms may stimulate regulatory cells of the immune system to inhibit inflammation and provide a natural defense against pathogenic species through competition [56,57].

Poor oral hygiene is strictly related to oral microbiota modifications, especially in subgingival communities. In this context, Gram-negative species (e.g., *Prevotella*, *Selenomonas*, and *F. nucleatum*) can significantly increase after 2–3 weeks of plaque accumulation, and clinical inflammation of the gingiva is a common clinical presentation of this condition [58]. On the other hand, the depletion of Gram-positive species (e.g., *R. dentocariosa*, *Propionibacterium*, and *S. maltophila*) has negative implications for oral health [58].

Furthermore, the development of periodontitis has been associated with the accumulation of different Gram-negative species compared to gingivitis. In 1998, Socransky et al. [59] identified “the red complex” including three different bacteria species (*Porphyromonas gingivalis*, *Tannerella forsythia*, and *Treponema denticola*) closely related to the arousal of clinical signs and symptoms of periodontitis, and strictly associated with the severity of the disease. In recent years, periodontitis has been correlated with several pathogens, such as *F. alocis*, *Porphyromonas*, *Synergistetes*, *Peptostreptococcaceae*, and *A. actinomycetemcomitans*, associated with aggressive periodontitis [60,61,62,63].

Moreover, a strict relationship has been hypothesized among members of the oral microbiome, which shows both antagonistic and synergistic interactions. For instance, *Fusobacterium nucleatum* was shown to increase the survivability of the periodontal pathogen, *P. gingivalis* [64], while *T. denticola* seems to benefit from the succinate produced by *P. gingivalis* [65]. Furthermore, *T. denticola* and *P. gingivalis* concentrations increase significantly in co-culture; indeed, alterations to glycine and glutamate catabolism by *T. denticola,* as well as changes to thiamine pyrophosphate and fatty acid synthesis by *P. gingivalis,* have been observed [63,66].

Thus, the recent scientific literature suggests potential correlations among oral microbiota and systemic diseases, probably due to the dissemination of pro-inflammatory, invasive, anaerobic, and oral pathogens into the gut [67,68,69,70,71,72].

## 3. Gut-Microbiota and Musculoskeletal Health: “Gut–Joint Axis”

Recent evidence has focused on the impact of gut microbiota on musculoskeletal health, highlighting the role of the “gut–joint axis” in the regulation of the pathogenic pathways of musculoskeletal conditions [25,26,73,74]. However, to date, the longevity has been negatively correlated with increased alpha diversity in gut microbiota, with recent research focusing on “leaky gut syndrome”, an aged-related condition characterized by increased gut permeability, resulting in microbial products spreading in the bloodstream and an increase in inflammatory states [74,75,76].

Although several questions are still open about the role of the leaky gut syndrome in the development and progression of osteoarthritis (OA), serum levels of bacterial metabolites might be correlated with joint degeneration in OA patients [23]. Inflammation serum markers in patients with OA are positively associated with bacterially produced lipopolysaccharides (LPS), supporting the hypothesis of a role of microbiota-induced systemic inflammation in several pathways underpinning the development of OA [77,78]. Therefore, these findings suggested that gut dysbiosis, especially in the elderly, might be strictly linked to OA pathogenesis, and the gut–joint axis might be a potentially modifiable cofactor to be targeted by a comprehensive therapeutic intervention [21,22,23,79]. In line with these findings, the expert consensus of the European Society for Clinical and Economic Aspects of Osteoporosis, Osteoarthritis and Musculoskeletal Diseases (ESCEO) has recently supported the hypothesis that gut microbiota alterations might be considered as hidden risk factors for the development and progression of OA [79]. In this context, it has been proposed that gut microbiota modulation might have positive effects on OA [25]. Furthermore, a recent study on mice underlined that probiotic administration might significantly reduce pro-inflammatory cytokine production in knee cartilage [80]. Similarly, a probiotic diet might be effective in modulating prostaglandin-endoperoxide synthase 2 (PTGS2) and transforming growth factor-beta (TGF-β), with intriguing implications for targeting low-grade systemic inflammation promoted by dysbiosis in patients with OA [80]. In accordance with these findings, gut microbiota might be crucially affected by dietary supplements, with several studies underlining the positive effects of nutraceuticals in promoting health status in older adults, particularly in the case of early diagnoses [23,81].

The main linking factor between gut microbiota and OA seems to be represented by low-grade chronic inflammation, supporting a new OA phenotype called “metabolic OA”, where several pro-inflammatory stimuli are associated with drastic changes in the composition of the intestinal microbiota [22]. Thus, aging might play a key role in intestinal microbiota composition, inducing reduced phyla diversity, a greater proportion of *Bacteroides* spp., and a distinct abundance of *Clostridium* groups [82].

In the last decade, a growing literature showed that the alteration of gut and oral cavity microbiota could have a detrimental impact on the pathogenesis of autoimmune and inflammatory joint diseases, such as OA and rheumatoid arthritis (RA) [21,22,23]. Increased inflammation is a relevant pathophysiological mechanism in RA and the potential correlation between serum levels of bacterial metabolites and joint degeneration is a crucial issue for future investigation [83,84].

Furthermore, several components of intestinal microbiota might affect host immunity, particularly in patients with autoimmune diseases such as RA [85,86,87]. The correlation between intestinal immune cell activation and arthritis is based on the potential migration of gut-derived immune cells to the joints, provoking an impairment in terms of differentiation of T cell types (i.e., Treg cells) involved in the pathogenesis of RA [86,87,88,89].

Specifically, it has been hypothesized that gut dysbiosis might be a mediator for inflammation in the temporomandibular joint (TMJ) by regulating the microglial activation in the trigeminal nociceptive system [90,91]. Moreover, it should be noted that TMJ inflammation commonly leads to temporomandibular disorders (TMDs), which are considered a sub-classification of musculoskeletal disorders commonly treated with conservative approaches [91,92,93]. Furthermore, RA might affect TMJ by causing disease-related symptoms, with a correlation between laboratory values of various inflammatory biomarkers causing rheumatic diseases and the progression of TMD [94,95,96,97,98,99].

Scher et al. [100] compared the composition of subgingival microbiota in patients with RA against controls and revealed that *Prevotella* and *Leptotrichia* species might characterize patients with RA. Meanwhile, distinct subgingival microbiota was found in RA patients without periodontal diseases, suggesting that changes in oral microbiota might be RA-specific [101,102,103,104]. Furthermore, it has been demonstrated that serum antibodies against *P. gingivalis* could increase during the preclinical phase, becoming stable after the diagnosis of RA [105,106]. Thus, an association between periodontal bacteria exposure and RA autoantibody development might represent an emerging research topic in the future [106].

A growing literature now seems to support the role of oral–gut microbiota in inflammatory conditions (i.e., OA, RA, and TMJ arthritis); however, several questions are still open in this field and future studies are needed to better characterize this concept.

## 4. Impact of Probiotics on Oral Microbiota and Periodontal Diseases

Probiotics are defined as living microorganisms that can have beneficial effects on the host when taken in sufficient doses [107]. They are available in several food products, such as yogurts, milk-based foods, powders, capsules, and oral solutions [108].

**Figure 2:**
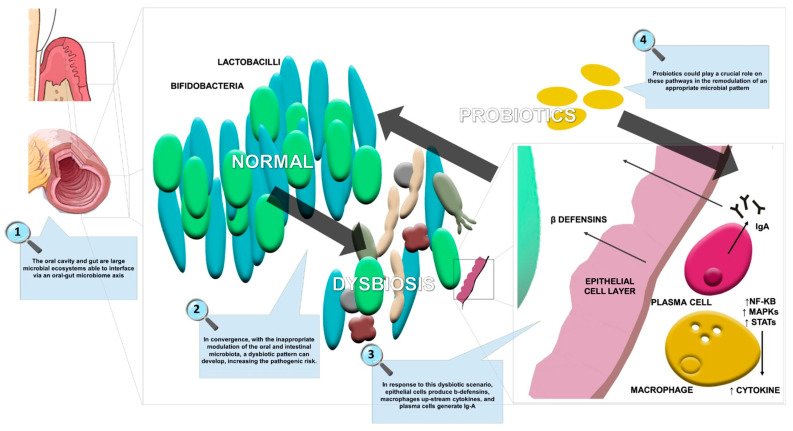
The effects of probiotics in oral and gut dysbiosis.

Moreover, the scientific literature is focused on the use of probiotic strains (e.g., *Lactobacillus*, *Bifidobacterium*, *Escherichia*, *Enterococcus*, and *B. subtilis*) and yeasts (e.g., *Saccharomyces*) in the maintenance of gastrointestinal microbiota balance. Figure 2 summarizes the effects of probiotics in oral and gut dysbiosis management, including restoration of the epithelial membrane resulting in a reduction of systemic inflammation.

Probiotic supplements with a concentration of 10^7^–10^8^ cells per gram could play a role in the treatment of inflammatory chronic diseases [107,108,109]. Indeed, recent evidence showed that probiotics might reinforce the epithelial barrier, thus allowing fibroblastic activity and epithelial cell migration [109,110].

Interestingly, probiotics that have a role in oral health are concentrated in the genera *Streptococcus*, *Lactobacillus*, *Bifidobacterium*, *Weissella*, *B. subtilis*, and *S. cerevisiae*, with their therapeutic use in dentistry growing significantly in recent years [111].

Several microorganisms isolated from the oral cavity are commercially produced as oral health-promoting probiotics, including *L. reuteri*, *L. brevis*, and *S. salivarius*, and their effectiveness is shown in the management of dental caries, oral candida infection, halitosis, and periodontal diseases [107,112,113,114,115].

Recently, Liu et al. [116] performed a systematic review and meta-analysis of randomized controlled trials (RCTs) on the effects of probiotics on gingival inflammation and oral microbiota composition in patients suffering from plaque-induced gingivitis. The authors included 11 RCTs with a total of 554 patients, reporting that the oral probiotics had no significant improvement in the Gingival Index (GI), Plaque Index (PI), and bleeding on probing (BOP) in patients affected by plaque-induced gingivitis. Moreover, no significant differences were found in the amount of *P. gingivalis*, *A. actinomycetemcomitans*, *P. intermedia*, and *F. nucleatum* between the probiotic group and the placebo group. Their findings were in line with another systematic review and meta-analysis by Hardan et al. [117] on the use of probiotics as an adjuvant therapy within clinical periodontal parameters. The authors showed that the use of probiotics did not improve the PI (*p* = 0.16). However, the systematic review also assessed the efficacy of probiotics as an adjuvant therapy in the treatment of periodontitis, and showed significant improvement in terms of PPD, CAL, and BOP (*p* < 0.001).

In line with these results, the effects of probiotics on the management of periodontal diseases were reported by several other studies [118,119,120]. Tekce et al. [119] evaluated the effectiveness of *L. reuteri* as an adjuvant treatment for chronic periodontitis patients, evaluating the clinical effects on periodontal tissues. The authors reported that plaque index, gingival index, bleeding on probing, and probing depth were significantly lower (*p* < 0.05) in the study group compared with controls at all time points [119].

In 2018, Invernici et al. [121] evaluated the effect of *Bifidobacterium animalis* subsp. *lactis* HN019 as an adjuvant to scaling and root planing (SRP) in patients with generalized chronic periodontitis (with 30% or more of the sites with probing pocket depth ≥ 4 mm and clinical attachment level ≥ 4 mm, and a minimum of five teeth with at least one site with CAL and PPD ≥ 5 mm). By collecting gingival crevicular fluid they determined the levels of IL-1β, IL-10, and IL-8. Furthermore, they evaluated the microbiota changes after probiotic therapy. The authors showed that subjects who underwent probiotic therapy reported higher levels of IL-10 than those at baseline at 30 days (*p* < 0.05) and showed greater amounts of *Actinomyces naeslundii* and *Streptococcus mitis,* and lower amounts of *P*. *gingivalis*, *T. denticola*, *F. nucleatum*, *C. showae*, and *E. nodatum* in deep periodontal pockets (*p* < 0.05) [121].

In 2020, the same research group evaluated the effects of *Bifidobacterium animalis* subsp. *lactis* HN019 in generalized chronic periodontitis patients [122]. They analyzed the immunocompetence of the gingival tissues by evaluating the expression of beta-defensin (BD)-3, toll-like receptor 4 (TLR4), and cluster of differentiation (CD)-57 and CD-4. Plaque accumulation, gingival bleeding, and the antimicrobial properties of HN019 were analyzed. Their results showed that subjects who underwent probiotic therapy presented with a lower PI at 30 days and had lower marginal gingival bleeding at 90 days (*p* < 0.05); in addition, increased BD-3, TLR4, and CD-4 expression in periodontal tissues were reported. Lastly, the findings showed a lower mean adhesion of *P. gingivalis* together with *B. lactis* HN019 to buccal epithelial cells (*p* < 0.05).

In this context, as depicted by Table 1, the oral microbiota could represent a potential target for probiotic supplementation to reduce the risk of periodontal diseases.

## 5. Role of Probiotics in Patients Affected by Arthritis

Probiotics could exert an immunomodulatory action by regulating intestinal inflammation and immune function and by preventing an increase in intestinal permeability and bacterial translocation. Therefore, probiotics might reduce the production of autoantibodies in the inflamed intestine and reduce the migration of pro-inflammatory immune cells from the gut tissue to the joints [123,124]. In this scenario, probiotics could be a beneficial intervention in the complex treatment of inflammatory joint diseases [125,126]. It should be noted that several studies [127,128,129,130,131,132,133,134,135,136,137] showed that the administration of specific probiotics (*E. faecium*, *L. casei*, *L. plantarum*, *B. longum, Bifidobacteria*, *P. histicola*, *L. acidophilus*, *L. helveticus*, *B. adolescentis*, and *L. fermentum*) may reduce RA symptoms by increasing anti-inflammatory cytokines (i.e., IL-10 and TGF-β) and inhibiting pro-inflammatory cytokines (i.e., IL-1β, IL-2, IL-6, IL-12, IL-17, and NF-κB), thus promoting the differentiation of CD4+ T cells into regulatory T cells (Tregs).

However, the role of probiotics in humans affected by inflammatory joint diseases is still debated in the scientific literature. In 2017, Mohammed et al. [124] performed a systematic review and meta-analysis of randomized or quasi-randomized clinical trials on the effect of probiotics on the treatment of RA. The authors included six randomized and controlled trials and three quasi-RCTs, with a total of 361 patients. Their results showed that oral probiotics lowered the pro-inflammatory cytokine IL-6 (SMD—0.708, 95% CI—1.370 to 0.047, *p* = 0.036), which is an indicator for joint destruction in RA, but no significant differences were found in disease activity score (DAS) and swollen joint count (SJC) between the probiotic and placebo groups.

Another recent systematic review with a meta-analysis performed by Zeng et al. [123] showed that the use of probiotics did not improve clinical variables such as DAS (*p* = 0.17) and swollen joint counts (*p* = 0.71) in patients. However, they assessed the efficacy of probiotics on inflammatory markers in RA and showed significantly lower levels of CRP (SMD −1.57 (−2.98, −0.15; *p* = 0.03)), highlighting the potential role of curcumin in CRP reduction [123]. Furthermore, Mandel et al. [127] demonstrated that the administration of *B. coagulans* to RA patients was effective in reducing the patient pain assessment score and the pain scale (*p* = 0.052 and 0.046, respectively). Moreover, a randomized, double-blind, placebo-controlled clinical trial showed the effects of *L. casei* on RA activity and inflammatory cytokines in women [128]. The authors demonstrated an improvement in the DAS (*p* < 0.01) associated with a reduction of serum levels, TNF-α, IL-6, and IL-12 (*p* < 0.05), and an increase of IL-10 (*p* < 0.05) in the group supplemented with probiotics [128]. Furthermore, Alipour et al. [129] treated patients with *L. casei* and found improvements in CRP levels, tender/swollen joint counts, and DAS28 compared to a placebo (*p* < 0.05). Zamani et al. [130] also demonstrated that patients who received a daily capsule containing three viable and freeze-dried strains (*L. acidophilus*, *L. casei*, and *B. bifidum*) showed an improvement in DAS28 (−0.3 ± 0.4 vs. −0.1 ± 0.4, *p* = 0.01) and serum high-sensitivity C-reactive protein (hs-CRP) concentrations (−6.66 ± 2.56 vs. +3.07 ± 5.53 mg/L, *p* < 0.001) compared with a placebo. Moreover, Cannarella et al. [131] demonstrated an exertive role of supplementing with a mixture of probiotics (*L. acidophilus, L. casei, L. lactis, B. lactis,* and *B. bifidus*) on TNF-α (*p* = 0.004) and IL-6 (*p* < 0.05), but no effect on DAS28 (*p* > 0.05). On other hand, a pilot study conducted by Hataka et al. [132] demonstrated that *Lactobacillus rhamnosus* administration did not show a statistically significant difference in the activity of RA in terms of both clinical variables and inflammatory markers. In accordance with this result, Pineda et al. [133] showed a non-significant decreasing trend in serum levels of IL-1α, IL-6, IL-10, IL-12, TNF-α, and Monocyte chemoattractant protein-1 (MCP-1) following *L. rhamnosus* combined with *L. reuteri* treatment in RA patients.

Concerning the use of probiotics in patients affected by spondyloarthritis, two studies affirmed no significant decrease in any disease activity markers such as the Bath Ankylosing Spondylitis Functional Index (BASFI) and Bath Ankylosing Spondylitis Disease Activity Index (BASDAI), ASAS-endorsed core domains, global health status, and CRP after probiotic intervention compared with a placebo (*p* > 0.05) [134,135]. As mentioned above, OA can be considered a persistent low-grade inflammation of the joints. Thus, over the past few years, there has been a growing interest in the role of the probiotics in OA therapy [136,137]. An important link between gut microbiota and patient’ clinical features highlighted the possibility of positively interfering with disease progression and presentation with microbiota modulation [26]. To date, mouse model studies on the use of probiotics, such as *C. butyricum*, *L. casei*, *L. acidophilus*, *L. fermentum*, *L. paracasei*, *S. thermophilus*, *B. longum*, *B. bifidum*, *B. breve*, *L. rhamnosus*, *L. plantarum*, *L. helveticus*, and *L. salivarius,* have demonstrated a positive role in the preservation of knee cartilage, synovial membrane, and fibrous tissue. Moreover, this supplementation significantly lowered serum levels of inflammatory and bone metabolism markers (such as metalloproteinases, cyclooxygenase-2, leukotriene B4, and cartilage oligomeric matrix protein) and inflammatory cytokines (such as IL-1β, IL-2, IL-6, IL-12, IL-17, TNF-α, and IFN-γ), while increasing levels of anti-inflammatory cytokines (IL-4 and IL-10) and anti-IFN-γ and glycosaminoglycans [138,139,140,141,142,143]. Meanwhile, a large RCT considered the effects of *L. casei Shirota* in human patients with knee OA who were asked to ingest either skimmed milk containing the probiotic or the placebo daily for 6 months. The study demonstrated an improvement in The Western Ontario and McMaster Universities Arthritis Index (WOMAC) in the intervention group (*p* < 0.05) [137].

Taken together, gut microbiome dysbiosis might be considered important in the pathogenic mechanism of inflammatory joint diseases both in terms of onset and progression; moreover, probiotics might play a role in the complex management of such chronic inflammatory diseases.

Accordingly, in Table 2 we describe the evidence for the role of probiotics in patients affected by musculoskeletal disorders.

## 6. Conclusions

This literature overview aimed to summarize the evidence on potential correlation among oral–gut microbiota, periodontal diseases, and arthritis, with an interest on the impact of probiotics on low-grade inflammation.

The scientific literature showed that poor oral hygiene can be correlated to oral microbiota modifications (e.g., *F. alocis*, *Porphyromonas*, *Synergistetes*, *Peptostreptococcaceae*, and *A. actinomycetemcomitans*) with a linkage between the accumulation of different Gram-negative species and the onset of periodontal diseases. Moreover, considering the role of the gut–joint axis in regulating molecular pathways involved in the pathogenesis of several musculoskeletal conditions (e.g., OA, RA, and TMJ arthritis), the microbiome may influence the pathogenesis of musculoskeletal diseases.

Although the pathophysiological mechanisms underpinning these interactions have not been fully characterized, a growing literature has been supporting the hypothesis of therapeutic action with dietary supplements and probiotics (e.g., *E. faecium*, *L. casei*, *L. plantarum*, *B. longum*, *Bifidobacteria*, *P. histicola*, *L. acidophilus*, *L. helveticus*, *B. adolescentis*, and *L. fermentum*) for the treatment of chronic inflammatory diseases.

In conclusion, in this paper, we described state-of-the-art findings from the scientific literature on the role of probiotics in the prevention and management of dysbiosis-related disorders. We consider that the gut–oral microbiota could be a new target for patients affected by periodontal diseases and arthritis in future.

However, it should be noted that there is still a gap in the scientific knowledge, not only on the role of oral microbiota in the pathogenesis of inflammation, but also on interactions among microbiota and other systemic conditions. Thus, further observational studies are needed to define, firstly, the specific target and, secondly, the impact of probiotics on patients affected by inflammatory diseases.

## Figures and Tables

**Table 1:** Impact of probiotics on the oral microbiota in human studies.

Authors	Journal and Year	Study Design	Study Sample	Intervention	Control	Outcomes	Main Findings
Yoo et al. [115]	Probiotics Antimicrob Proteins 2019	Systematic review and meta-analysis	Halitosis 3 RCT N = 86	Oral probiotics	/	Organoleptic scores; Volatile sulfur compounds concentration	Organoleptic scores were significantly lower in interventions (SMD = −1.93, 95% CI −2.85 to −1.02, *p* < 0.0001). Volatile sulfur compound concentration did not show significant differences (SMD = −0.02, 95% CI −2.12 to 2.07, *p* = 0.98). Probiotics showed a moderate effect on halitosis regarding organoleptic scores. The available evidence is insufficient for further recommendations.
Liu et al. [116]	Oral Dis 2022	Systematic review and meta-analysis	Plaque-induced gingivitis 11 RCT N = 554	Oral probiotics	/	GI, PI, and BOP; Inflammation biomarkers; Oral microecological environment	Interventions had no significant improvement in GI, PI, and BOP in any comparison (*p* = NS). No significant difference in intergroup analysis was found in volumes of gingival crevicular fluid, concentration of IL-1β, and counts of *Aggregatibacter actinomycetemcomitans*, *Porphyromonas gingivalis*, *Prevotella intermedia*, and *Fusobacterium nucleatum* (*p* = NS).
Hardan et al. [117]	Pharmaceutics 2022	Systematic review and meta-analysis	Periodontal disease 21 RCT N = 1089	Oral probiotics	/	PI, BoP, PPD, and CAL	The interventions did not improve the PI (*p* = 0.16). The interventions improved significantly PPD, CAL, and BoP compared to controls (*p* < 0.001, *p* < 0.001, and *p*= 0.005, respectively). Probiotics might be implemented to lead to an improvement in PPD, CAL, and BoP.
Seminario-Amez et al. [118]	Med Oral Patol Oral Cir Bucal 2017	Systematic review	Treatment and/or prevention of an infectious oral disease 12 RCT 2 meta-analyses 1 systematic review	Oral probiotics	/	BoP, GI, and PD; Oral microecological environment	Decrease in colony-forming unit counts of cariogenic pathogens (*S. mutans*). The studies included in the review reported a clinical improvement of BoP, PD, and GI, but no significant difference in colony-forming unit counts of periodontal pathogens.
Tekce et al. [119]	J Clin Periodontol 2015	RCT	Patients with chronic periodontitis patients N = 40	Lozenges containing *L. reuteri* + SRP	Placebo + SRP	PI, BoP, GI, and PD	BoP, PI, GI, and PD were significantly (*p* < 0.05) lower in IG compared with CG at all time points. *L. reuteri*-containing lozenges might slow recolonization and improve clinical outcomes of chronic periodontitis.
Invernici et al. [121]	J Clin Periodontol 2018	RCT	Patients with chronic periodontitis N = 41	*Bifidobacterium animalis* subsp. *lactis* (*B. lactis*) HN019-containing probiotic lozenges + SRP	Placebo + SRP	PI, BoP, PPD, CAL, GR; Gingival crevicular fluid levels of inflammation biomarkers; Oral microecological environment	In moderate and deep pockets, the IG had larger CAL gain and lower PPD than the CG at 90 days (*p* < 0.05). Overall, PI, BoP, and GR were not significant at 90 days (*p* = NS). IG reported higher intragroup levels of IL-10 at 30 days (*p* < 0.05). IG exhibited a larger count of *A. naeslundii* and *S. mitis* and greater reduction in *P. gingivalis, T. denticola, F. nucleatum vincentii, C. showae,* and *E. nodatum* compared to CG (*p* < 0.05) for deep periodontal pockets. The use of *B. lactis* HN019 as an adjunct to SRP promotes additional clinical, immunological, and microbiological benefits.
Invernici et al. [122]	PLoS One 2020	RCT	Patients with chronic periodontitis N = 30	*B*. *lactis* HN019 probiotics + SRP	Placebo + SRP	PI, BOMP, expression of beta-defensin (BD)-3, toll-like receptor 4 (TLR4), cluster of differentiation (CD)-57 and CD-4, IgA saliva levels. Antimicrobial properties.	IG presented lower PI at 30 days and lower BOMP at 90 days when compared with CG (*p* < 0.05). No significant changes were observed in IgA levels (*p* > 0.05). Healthy sites of IG had significantly higher BD-3 and TLR4 immunoreactivity at 30 days when compared to baseline (*p* < 0.05). The IG exhibited significantly higher BD-3 and TLR4 expressions on diseased sites at 30 days when compared to CG (*p* < 0.05). CD-57 analysis showed no significant differences (*p* = NS). IG showed a significantly greater immunoreactivity for CD-4 at 30 days when compared with baseline (*p* < 0.05). *B. lactis* HN019 reduced the adhesion of *P. gingivalis* to buccal epithelial cells. *B. lactis* HN019 might be effectively used in non-surgical periodontal therapy.

Abbreviations: BoP: bleeding on probing; BOMP: bleeding on marginal probing; CAL: clinical attachment level; CG: control group; CI: confidence interval; GI: gingival indices; GR: gingival recession; IG: intervention group; N: number; NS: not significant; PD: probing depth; PPD: probing pocket depth; PI: plaque index; SMD: standardized mean difference; SRP: scaling and root planing; RCT: randomized controlled trial.

**Table 2:** Probiotics for musculoskeletal disorders in human studies.

Authors	Journal and Year	Study Design	Study Sample	Intervention	Control	Outcomes	Main Findings
Zeng et al. [123]	Front Immunol 2022	Systematic review and meta-analysis	RA 10 RCT N = 632	Oral probiotics	/	DAS-28, SJC, TJC, CRP	There was a statistical difference between the experimental group and the control group in CRP decrease (SMD −1.57 (−2.98, −0.15; *p* = 0.03)). No significant improvement in DAS28, SJC, and TJC was found (*p* = NS).
Mohammed et al. [126]	Clin Rheumatol 2017	Systematic review and meta-analysis	RA 6 RCT 3 Q-RCT N = 361	Oral probiotics	/	DAS, SJC, TJC; cytokines (TNF-α); interleukin (IL-1β, IL-6, IL-10, IL-12, and); Inflammation biomarkers (CRP)	Probiotics lowered the pro-inflammatory cytokine IL-6 (SMD − 0.708, 95% CI − 1.370 to 0.047, (*p* = 0.036)). Probiotics showed no improvement in clinical variables compared to placebo. The available evidence is insufficient for further recommendations.
Mandel et al. [127]	BMC Complement Altern Med 2010	RCT	RA N = 45	*B. coagulans*	Placebo	Pain examination, ACR criteria, HAQ, CRP,	Statistically significant improvement in the patient pain assessment score (*p* = 0.052) and statistically significant improvement in pain scale (*p* = 0.046) in intervention group.
Vaghef-Mehrabany et al. [128]	Nutrition 2014	RCT	RA N = 46	*L. casei*	Placebo (maltodextrin)	DAS-28; cytokines (TNF-α); interleukins (IL-6, IL-12, IL-10)	Disease activity score was significantly decreased by the intervention (*p* < 0.01). TNF -α, IL-6, and IL-12 significantly decreased in the probiotic group (*p* < 0.05); serum level interleukin-10 was increased with supplementation (*p* < 0.05).
Alipour et al. [129]	Int J Rheum Dis 2014	RCT	RA N = 46	*L. casei*	Placebo	SJC, TJC, DAS28; cytokines (TNF-α), IL-1β, IL-6, IL-10, IL-12	*L. casei* decreased serum level of CRP, tender and swollen joint counts, global health (GH) score and DAS28 (*p* < 0.05). A significant difference was observed between the two groups for IL-10, IL-12, and TNF-α changes through the study course (*p* < 0.05) in favor of the probiotic group. No adverse effects were reported for the intervention.
Zamani et al. [130]	Int J Rheum Dis 2016	RCT	RA N = 60	*L. acidophilus*, *L. casei*, *B. bifidum*	Placebo	DAS28, SJC, TJC; CRP	Probiotic supplementation improved DAS28 (−0.3 ± 0.4 vs. −0.1 ± 0.4, *p* = 0.01) and serum high-sensitivity C-reactive protein (hs-CRP) concentrations (−6.66 ± 2.56 vs. +3.07 ± 5.53 mg/L, *p* < 0.001).
Cannarella et al. [131]	Nutrition 2021	RCT	RA N = 42	*L. acidophilus*, *L. casei*, *L. lactis*, *B. lactis*, *B. bifidum + maltodextrin*	Placebo + maltodextrin	DAS28; TNF-α, interleukin (IL-6, IL-10), CRP	Probiotics improved white blood cell counts, TNF-a (*p* = 0.004) and IL-6 plasma levels (*p* < 0.05). No effects were found in DAS28 (*p* > 0.05).
Hataka et al. [132]	Scand J Rheumatol 2003	RCT	RA N = 21	*L. rhamnosus*	Placebo	SJC, TJC, HAQ, cytokines (TNF-α, MPO), Interleukin (IL-1α, IL-1β, IL-6, IL-8, IL-12, IL-10), CRP, ESR	No significant improvements were found in all the outcomes (*p* > 0.05).
Pineda et al. [133]	Int Med J Exp Clin Res 2011	RCT	RA N = 29	*L. rhamnosus*, *L. reuteri*	Placebo	SJC, TJC; cytokines (TNF-α, GM-CSF, G-CSF, IL-17, sCD40 ligand, MIP-1α, MIP-1β, MCP-1), interleukin (IL-1α, IL-1β, IL-6, IL-8, IL-12p70, IL-15, IL-10,); CRP, ESR	There were no statistically significant differences between groups in clinical variables (*p* > 0.05). There was a trend for reduced secretion of pro-inflammatory cytokines, especially GM-CSF, IL-1α, IL-6, IL-15, and TNF-α, following probiotic treatment compared to placebo.
Jenks et al. [134]	J.Rheumatol 2010	RTC	Spondyloarthritis N = 63	*S. salivarius*, *B. lactis*, *L. acidophilus*	Placebo	BASDAI, BASFI, ASAS-endorsed core domains; CRP	No significant improvements were found in any outcome (*p* > 0.05).
Brophy et al. [135]	BMC Musculoskelet Disord 2008	RCT	Spondyloarthritis N = 147	*L. salivarius*, *L. paracasei*, *B.* *Infantis*, *B. bifidum,*	Placebo	Global wellbeing (0–10 scale), Disease activity (0–10 scale) and Function (0–10 scale)	No significant improvements were found in any outcome (*p* > 0.05).
Lei et al. [137]	Benef Microbes 2017	RCT	Knee OA N = 215	*L. casei Shirota*	Placebo	WOMAC, VAS, CRP	Patients in the probiotic group had significantly improved WOMAC and VAS scores, and decreased serum hs-CRP levels (*p* < 0.05).

Abbreviations: RA: Rheumatoid Arthritis; DAS: disease activity score; SJC: swollen joint count; TJC: tender joint count; CRP: C-reactive protein; ACR: American College of Rheumatology; HAQ-DI: Health Assessment Questionnaire Disability Index; BASDAI: Bath Ankylosing Spondylitis Disease Activity Index; BASFI: Bath Ankylosing Spondylitis Functional Index; ASAS: Assessment in Ankylosing Spondylitis; WOMAC: Western Ontario and McMaster Universities Arthritis Index; VAS: Visual Analogue Scale; OA: Osteoarthritis.

## Data Availability

Not applicable.
